# Nutrition and Hydration at the End of Life in Intensive Care and General End-of-Life Care Settings: Balancing Clinical Evidence, Patient-Centered Care, and Ethical and Legal Principles—A Narrative Review

**DOI:** 10.3390/nu17233705

**Published:** 2025-11-26

**Authors:** Mircea Stoian, Adina Stoian, Claudia Bănescu, Sergio Rares Bandila, Dragoș-Florin Babă, Leonard Azamfirei

**Affiliations:** 1 Department of Anesthesiology and Intensive Care Medicine, George Emil Palade University of Medicine, Pharmacy, Science and Technology of Târgu Mureș, 540103 Târgu Mureș, Romania; mircea.stoian@umfst.ro (M.S.); leonard.azamfirei@umfst.ro (L.A.); 2 Department of Pathophysiology, George Emil Palade University of Medicine, Pharmacy, Science and Technology of Târgu Mureș, 540136 Târgu Mureș, Romania; 3 Department of Genetics, George Emil Palade University of Medicine, Pharmacy, Science and Technology of Târgu Mureș, 540142 Târgu Mureș, Romania; claudia.banescu@umfst.ro; 4 Orthopedic Surgery and Traumatology Service, Marina Baixa Hospital, Av. Alcade En Jaume Botella Mayor, 03570 Villajoyosa, Spain; sergiob1976@gmail.com; 5 Department of Cell and Molecular Biology, George Emil Palade University of Medicine, Pharmacy, Science and Technology of Târgu Mureș, 540142 Târgu Mureș, Romania; dragos-florin.baba@umfst.ro

**Keywords:** nutrition, end-of-life care, medical and ethical dilemma, personalized nutrition, cachexia, narrative review

## Abstract

**Background/Objectives**: Nutrition at the end of life raises many dilemmas. “End of life” refers to the period associated with a progressive incurable disease, with a life expectancy of less than six months, and limited curative treatments. In intensive care units (ICUs), decisions about artificial nutrition and hydration (clinically assisted nutrition and hydration, CANH) are especially complex because patient goals shift from survival to comfort. Nutrition and hydration are often requested by patients and their families, even when clinical benefits are uncertain. This article aims to provide a multidimensional analysis of the pathophysiological, clinical, ethical and legal considerations of nutritional support in the final stages of life. **Methods**: We conducted a narrative review of the literature published between January 2000 and June 2025 by searching the PubMed/MEDLINE, Web of Science, and Scopus databases and included original articles, clinical trials, reviews, international guidelines, and public policy documents involving adult population at the end of life. The narrative approach enabled the multidimensional integration of the collected data. **Results**: Terminally ill patients often develop anorexia and cachexia leading to irreversible muscle loss and resistance to nutritional support. CANH (enteral or parenteral) has limited success and carries increased risks. In advanced cancer and dementia, studies do not show clear benefits for survival or quality of life. Nutritional counseling and oral supplements may help alleviate symptoms, but manual feeding remains the standard of care in the terminal stages. In ICU settings, starting or maintaining CANH demands careful evaluation of goals, prognosis, and burdens. Cultural legal differences and approaches between countries also influence clinical practice and family expectations. **Conclusions**: CANH at the end of life should be viewed as a medical intervention that requires both scientific and ethical justification. The decision to initiate or discontinue it should be individualized. Clear and empathetic communication between the medical team, patient, and family is essential to avoid inappropriate decisions.

## 1. Introduction

The end of life is a natural and inevitable stage of human existence. Death is a universal phenomenon and an integral part of the human condition. However, the term “end of life” is often used in clinical practice to refer to the period when a person faces an incurable, progressive illness, with a limited life expectancy of less than six months [1]. This period is marked by functional decline, reduced autonomy, and a focus on patient comfort rather than curative treatments [2,3].

Care should be personalized considering the progression of the disease, the level of awareness, and the patient’s remaining autonomy [4]. Accordingly, the care of terminally ill cancer patients emphasizes pain control, symptom (e.g., nausea, agitation, and anxiety) management, and psychological and spiritual support [5].

Recognizing the disease trajectory in cases of terminal illnesses or during the end of life is vital for planning interventions and making appropriate medical and ethical decisions [6]. Nutrition holds great symbolic and emotional value for the patient and their family; thus, there may be persistent requests to continue CANH even when it offers no therapeutic benefits [7]. The term artificial nutrition and hydration is used in a strictly medical sense, referring to enteral and/or parenteral nutrition as essential therapeutic interventions when oral feeding is no longer possible or safe, in line with international guidelines (ESPEN, ASPEN) [8,9]. In the international literature and in specific ethical and legal frameworks (e.g., NICE, EAPC) [10,11,12], this concept aligns with that of clinically assisted nutrition and hydration (CANH).

This paper offers a narrative review of the implications and debates surrounding the use of nutritional support in the final stages of life, both in intensive care units (ICUs), where proportionality and time-limited trials of CANH are often discussed, and in general end-of-life care settings [13,14]. A narrative synthesis was selected because it enables the combination of diverse sources, including clinical studies, pathophysiological evidence, professional guidelines, legal texts, and jurisdiction-specific legal frameworks, which cannot be effectively combined through meta-analysis [13,14,15,16,17]. The numerous available studies, from clinical and pathophysiological research to ethical, legal, and socio-cultural investigations, cannot be uniformly summarized in a meta-analysis or systematic review. However, the narrative approach enables the multidimensional integration of these aspects, providing a comprehensive view of the topic without the rigidity of a systematic review. This narrative review connects clinical evidence with bedside decision-making frameworks and jurisdiction-specific ethical and legal considerations, targeting clinicians who need to make proportionate, patient-centered decisions about nutrition and hydration near the end of life. Therefore, nutrition at the end of life raises many medical, ethical, and psychosocial questions.

## 2. Materials and Methods

This narrative review used a transparent, open process to ensure methods were understandable and repeatable.

Search strategy: PubMed/MEDLINE, Embase, Scopus, and Web of Science were searched for publications from January 2000 to June 2025. The search used Medical Subject Headings (MeSH) and free-text keywords related to “end-of-life care,” “artificial nutrition,” “parenteral nutrition,” “enteral nutrition,” “feeding tube,” “hydration,” “ethics,” and “law.” A sample PubMed search string was: (“end of life” OR “palliative care”) AND (“artificial nutrition” OR “enteral nutrition” OR “parenteral nutrition” OR “feeding tube”) AND (“ethics” OR “law” OR “decision-making”). Reference lists of key reviews, guidelines, and position statements were hand-searched for additional sources.

Eligibility criteria: We included original research articles, narrative or systematic reviews, clinical guidelines, position statements, and ethical or legal policy documents related to adult end-of-life populations. Exclusion criteria were: pediatric or preclinical studies, articles not related to nutrition or hydration decisions, and publications in languages other than English.

Selection process: Two reviewers independently screened titles and abstracts for relevance. Disagreements were resolved through discussion. Approximately 260 records were identified, and 160 publications, including 16 guidance or legal documents, were selected for full-text review and included in the narrative synthesis (Supplementary Figure S1).

Synthesis approach: Data were analyzed thematically, aligning findings with key clinical decision points, physiological mechanisms, outcomes (such as survival, comfort, aspiration, and quality of life), and ethical or legal frameworks. Results were summarized narratively due to the heterogeneity of study designs and evidence sources.

Since this is a narrative review, no meta-analysis was performed, and no formal quality appraisal tool (e.g., GRADE, ROBIS) was used. The methodological transparency was maintained through a structured process of selection and synthesis. A formal PRISMA diagram was not applicable; instead, a concise numerical flow summary is provided. Certainty labels (high, moderate, low) were assigned pragmatically based on the hierarchy of sources (international guidelines > systematic reviews > primary studies), study design, consistency of findings across publications, effect size directionality, and biological plausibility.

## 3. Context and Definitions of Nutrition for End-of-Life Patients

The basal metabolic rate drops markedly as life approaches its end [18]. The human body experiences substantial physiological changes that diminish its ability to digest, absorb, and metabolize nutrients [19]. Factors such as systemic inflammation, metabolic dysfunction, and declining organ function influence these changes. This metabolic decline is accompanied by reduced enzymatic activity and intestinal absorptive capacity [20]. Changes in taste and smell occur as a result of cytotoxic treatments. Dysphagia and the loss of the swallowing reflex are common, increasing the risk of aspiration and complicating nutrition [11,21]. Furthermore, patients in the terminal stages may exhibit altered states of consciousness that impair their ability to feed [11,21].

The Warburg effect, which enhances aerobic glycolysis and lactate production in tumor cells, provokes an energy imbalance. Tumors consume large amounts of glucose and deplete the host’s energy reserves, with lactate recycled by the liver into glucose at an additional energy cost. This process promotes tumor growth and the degradation of host tissues [22].

Malnutrition involves imbalances in energy and nutrient intake, such as weight loss, micronutrient deficiencies, and overweight or obesity, with related complications including cardiovascular diseases and type 2 diabetes [23,24]. Nutritional deprivation caused by comorbidities that hinder adequate dietary intake leads to the depletion of physical reserves, as seen in patients in the terminal stages of a disease [25,26].

Anorexia is the loss of the desire to eat, common in advanced chronic diseases [27]. Terminal anorexia is complex, resulting from the interaction of several pathophysiological factors, and is an essential part of cachexia [28]. In elderly patients and those with advanced chronic diseases, the condition is worsened by systemic inflammation mediated by proinflammatory cytokines, such as interleukin-1 (IL-1), interleukin-6 (IL-6), tumor necrosis factor-alpha (TNF-α), interferon-gamma (IFN-γ), or C-reactive protein (CRP) [29]. These substances directly affect the appetite-regulating centers in the hypothalamus, decreasing the desire to eat and accelerating protein breakdown [30,31].

Cachexia, derived from the Greek words kakos, meaning “bad,” and hexis, meaning “condition,” refers to the progressive loss of fat and muscle mass caused by increased proteolysis, decreased protein synthesis, and enhanced lipolysis, which together increase mortality [27]. Unlike simple caloric restriction, cachexia causes severe and irreversible weight loss regardless of nutritional support. Cachexia is observed in advanced cancer, chronic infections, and long-term illnesses, including COPD, kidney and heart failure, tuberculosis, HIV, autoimmune diseases, and advanced conditions where ongoing systemic inflammation, metabolic issues, and hormonal changes promote and trigger catabolic processes [32,33,34]. It should be noted that many studies investigating the hormonal mechanisms underlying appetite regulation and cachexia use animal models, which may limit the direct extrapolation of these findings to human patients. IL-6 serum levels are notably higher in cachectic cancer patients experiencing weight loss than in non-cachectic patients or healthy individuals [35]. Cachexia differs from starvation caused by systemic inflammation and severe metabolic disturbances, which lead to fat becoming the primary energy source and result in a substantial energy deficit [33,36].

The loss of the desire to eat in anorexia occurs during severe caloric deprivation, a common symptom in terminal cancers. Anorexia–cachexia syndrome (ACS) is a clinical condition in which anorexia occurs alongside cachexia. ACS is characterized by weight loss, decreased appetite, early satiety, muscle wasting, and weakness [35]. In cancer, the tumor is the leading cause, along with the effects of chemotherapy and radiotherapy [36,37]. Studies on animals have demonstrated that IL-1 influences signaling in the hypothalamus, altering the duration, frequency, and size of food portions ingested by rats. The mechanism for ACS is the increase in serotonin levels through the tryptophan pathway, responsible for activating proopiomelanocortin neurons (POMC), which were found to be involved in anorexia in tumor-bearing animals [37,38]. Nonetheless, serum cytokine levels do not necessarily correlate with anorexigenic effects, as they may act in a paracrine or autocrine manner [39].

## 4. Pathophysiology of Reduced Food Intake at the End of Life

Cachexia results from a complex interplay of pathophysiological factors involving peripheral and central mechanisms, along with hormonal, genetic, and metabolic factors.

### 4.1. Peripheral Mechanisms of Anorexia

Proinflammatory cytokines secreted by tumors, macrophages, and other immune cells exert peripheral effects that decrease food intake and cause weight loss by disrupting carbohydrate, lipid, and protein metabolism. These substances promote proteolysis and lipolysis through factors such as the proteolysis-inducing factor (PIF) and lipid-mobilizing factor (LMF), which increase peripheral catabolism and induce insulin resistance [37].

The release of lactate, parathyroid-like peptide, and other substances by tumors affects energy metabolism and appetite. Gastrointestinal dysfunctions (gastroparesis or dysphagia) or digestive obstructions caused by neoplasms are also involved. Furthermore, micronutrient (e.g., zinc) deficiency and tumor hypoxemia can contribute to anorexia [35,40].

In addition to inflammation, the increase in peripheral tryptophan levels affects central serotonin, as it has an anorexigenic effect. Substantial hormonal changes occur, including an increase in peptide YY and a decrease in ghrelin secretion, as well as an increase in leptin and cholecystokinin (CCK) levels [28,32].

Cancers that affect the gastrointestinal tract may cause discomfort and pain during or after eating, hampering nutrient absorption [41]. The pain from the disease and the discomfort from chemo and radiotherapy can lead to the refusal of food and hydration. Thus, managing these individuals represents a challenge for staff in oncology clinics and palliative care [42]. Dyspnea, pain, nausea, and xerostomia are other peripheral factors that restrict food intake in the terminal stages of the disease [13].

### 4.2. Genetic Determinants of Anorexia–Cachexia

In addition to metabolic and inflammatory mechanisms, genetic and molecular factors have been reported to modulate the susceptibility and severity of anorexia–cachexia [43,44]. Single-nucleotide polymorphisms (SNPs) in proinflammatory cytokine genes (IL-1, IL-6, and TNF-α) are associated with variability in the systemic inflammatory response. This may partly explain inter-individual differences in the severity of clinical manifestations [45]. Variants in genes involved in the regulation of energy balance and appetite pathways, such as melanocortin-4 receptor (MC4R), may interfere with anorexigenic signaling and resistance to neuropeptide Y. Alterations in the growth hormone/insulin-like growth factor-1 (GH/IGF-1) axis have also been implicated in intensified muscle wasting [46]. Moreover, mutations in tumor-driver genes (e.g., TP53, MYC, and HIF1A) facilitate metabolic reprogramming and promote the Warburg effect, further worsening the energy imbalance [46]. Hence, integrating genetic and molecular data with clinical and metabolic profiles may improve risk stratification and enable personalized interventions, particularly when combined with artificial intelligence-based models.

### 4.3. Central Mechanisms of Anorexia

At the level of the central nervous system (CNS), inflammation and tumors affect hypothalamic neurotransmission. Circulating cytokines can directly influence neurons in the CNS. Although cytokines are too large to cross the blood–brain barrier (BBB) by simple diffusion [47], they can access the CNS at structures like the median eminence and the arcuate nucleus (ARC) through fenestrated capillaries. Notably, the ARC is a key center for integrating leptin and, potentially, cytokine signals [48]. Additionally, active transport mechanisms across the BBB and blood–cerebrospinal fluid (CSF) barrier exist for leptin and proinflammatory cytokines, indicating that brain regions behind the BBB may also respond to inflammatory signals involved in cachexia [49].

Leptin, a hormone-like protein discovered in 1994, is an adipokine with hormonal functions, primarily produced by adipose tissue and the gastric mucosa. Leptin is a negative regulator of body mass and appetite, and is closely related in structure and function to IL-6 [50]. In the CNS, leptin crosses the BBB and acts on the hypothalamus to signal satiety and reduce food intake [6]. Leptin promotes satiety, regulates energy balance, and interacts with insulin, affecting glucose and lipid homeostasis [51]. Serum leptin levels increase after a meal, decrease during fasting, and are directly proportional to adipose tissue mass [52,53]. Peripherally, leptin increases vagal activity, slows gastric emptying, stimulates the intestinal absorption of carbohydrates and proteins, decreases lipid levels, reduces the release of apolipoproteins into circulation, and promotes fatty acid oxidation in the liver and muscle, preventing ectopic fat buildup [54,55,56]. When fat stores decrease, leptin levels drop and appetite rises, a vital survival mechanism [51,57]. In patients with inflammatory diseases (e.g., cancer), leptin levels are typically low; however, they do not increase appetite because of hypothalamic disorders and excess proinflammatory cytokines [58,59].

Between 2012 and 2022, 553 patients with advanced cancer and cachexia were prospectively enrolled and treated with an anti-cachectic combination of megestrol acetate, celecoxib, L-carnitine, and antioxidants. The results showed that changes in leptin levels were inversely related to inflammatory markers; thus, they reflected improvements in body composition, energy metabolism, muscle strength, functional performance, and quality of life (QoL). In a multivariate analysis, changes in leptin were used as an independent predictor of increases in lean mass, muscle index, and grip strength, and decreases in energy expenditure. The predictive ability of leptin surpassed that of other inflammatory markers [60].

Ghrelin is a hormone produced by the stomach in response to hunger. Ghrelin is also a growth hormone-releasing peptide that stimulates AgRP/NPY neurons and reduces the production of proinflammatory cytokines in immune and endothelial cells [61,62,63]. Plasma ghrelin levels are often elevated in patients with cachexia as a compensatory response to energy imbalance, although they may decrease in advanced cancer [64]. The administration of exogenous ghrelin in animals with tumors, chronic renal failure, or heart failure was reported to decrease anorexia, increase muscle mass, and enhance mitochondrial oxidative capacity, indicating central and peripheral anti-catabolic effects [65,66].

Thus, ghrelin and leptin are two hormones with opposite effects on appetite regulation. In cancer, unlike starvation, reduced leptin and elevated ghrelin do not increase appetite, indicating a central neurohormonal imbalance that might explain cancer-associated anorexia [67,68]. Proinflammatory cytokines influence this process: TNF-α decreases leptin secretion, but hypothalamic leptin receptors are overexpressed; additionally, cytokines inhibit gastric ghrelin secretion [69].

Inflammatory cytokines can also influence neuronal activity by stimulating prostaglandin synthesis at the BBB, specifically in endothelial cells and perivascular macrophages. These mediators are small, lipid-soluble molecules able to diffuse through the BBB [70]. During inflammation, cytokines rapidly activate endothelial cells, which send signals to neurons via prostaglandins, particularly PGE2. Notably, the enzymes responsible for the synthesis of PGE2 are highly expressed [71]. On the other hand, the pharmacological blockade of cyclooxygenase (COX) reduces IL-1β- and LPS-induced anorexia. Furthermore, COX inhibitors were reported to lessen anorexia and weight loss in tumor-bearing animals [72,73].

The hypothalamic central melanocortin system is another key target of inflammatory signaling in the CNS. POMCs, located in the ARC and the nucleus of the solitary tract, produce the precursor peptide POMC [74]. POMC is cleaved into several bioactive peptides, such as the alpha-melanocyte-stimulating hormone (α-MSH). α-MSH is a potent anorexigenic mediator that acts through MC4R, which is widely expressed in the CNS, leading to decreased appetite and increased energy expenditure [49]. Moreover, cytokines enhance POMC expression and decrease food intake, an effect hindered by blocking melanocortin receptors [75].

The orexigenic neuropeptide Y (NPY) promotes food intake and causes obesity. Furthermore, NPY is dysregulated in cachexia: inflammation and cytokines (e.g., IL-1β) decrease its expression and release in the hypothalamus, resulting in functional resistance to this peptide [49,76]. Therefore, cancer-related anorexia caused by NPY deficiency cannot be treated only by administering NPY; instead, downstream signaling issues should also be addressed [49].

The serotonin system helps regulate food intake and energy metabolism; its overall activation exerts a suppressive effect [77]. The 5-HT2C receptors found on POMCs in the hypothalamus promote appetite suppression and regulate energy metabolism. In tumor-bearing animals, serotonin levels increase in the ventromedial hypothalamic nucleus, promoting anorexia [78]. Thus, blocking serotonin receptors in this area improves food intake. Specific serotonin receptor subtypes also help regulate autonomic functions, such as gastrointestinal motility [77,78].

**Figure 1:**
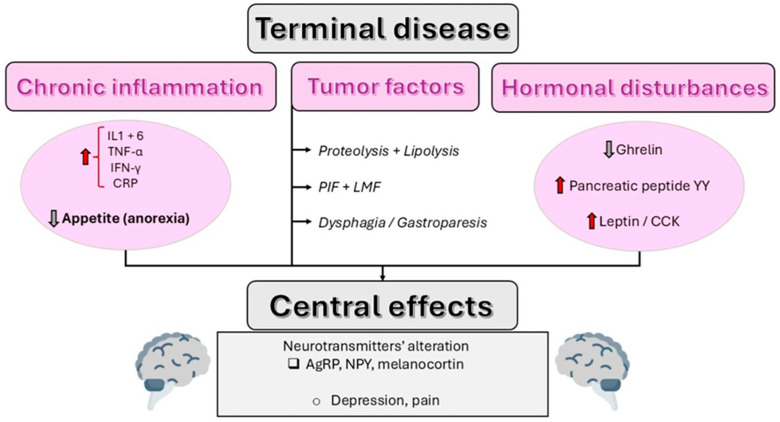
Pathophysiology of decreased food intake at the end of life. General note: All figures are original schematics created by the authors to illustrate the concepts discussed in this review. No previously published material was adapted or reused.

Figure 1 illustrates the pathophysiological processes involved in anorexia and cachexia associated with terminal illness.

In conclusion, understanding the complex mechanisms involved in anorexia and cachexia can help decide when to start CANH. The abovementioned inflammatory and neuroendocrine processes influence the body’s resistance to nutritional intake, and thus explain why CANH often has little effectiveness in the terminal stages.

## 5. Clinical Implications and Considerations

Decreased energy needs, vitamin and mineral deficiencies, and reduced muscle mass are common in advanced age. Associated psychosocial factors, such as depression, social isolation, loneliness, feelings of abandonment, cognitive decline, and a lack of social support networks, further worsen this decline [79].

Nutritional support is essential for maintaining the nutritional status of critically ill patients who are unable to receive oral nutrition. Such supplementation is provided either enterally, through nasogastric or gastrostomy tubes, or parenterally, through intravenous infusions, depending on the patient’s condition and their digestive system’s ability to process nutrients [14,80].

Gastrointestinal motility disorders, such as gastroparesis and intestinal stasis, can exacerbate the sensation of fullness and postprandial discomfort, ultimately leading to a reluctance to eat. In the final stages of the disease, weight loss (including adipose tissue, muscle mass, and sarcopenia) is common [81,82]. The primary objective is thus to manage pain and discomfort, rather than correcting nutritional deficiencies, although this is not entirely excluded [83,84].

In patients with advanced dementia, the end of life involves a complete loss of autonomy, swallowing difficulties, recurrent infections, and malnutrition [85]. Such patients often refuse to eat, appear disinterested, and are confused. A Cochrane review found no evidence that enteral feeding is beneficial for them; however, it remains widely used in the care of these patients [86], often at the family’s request. Careful manual feeding is considered the standard of care [15]. Thus, care centers on comfort, preventing suffering, avoiding invasive procedures, such as probes and infusions, and unnecessary hospitalizations. Additionally, CANH, antibiotics, and resuscitation are generally discouraged, although this depends on the patient’s values and preferences, if these are known [85].

Nutritional interventions should be personalized, considering the limited benefits of CANH in the terminal stages, the associated risks (including aspiration, infections, and discomfort), and the patient’s preferences [81,82]. A tailored nutritional plan can help prevent complications related to undernutrition or overnutrition. However, it does not apply to some patients who no longer benefit from curative nutritional therapy but palliative dietary treatment. Therefore, the nutritional plan should be continuously assessed and adjusted to meet the changing needs of patients [14]. In the ICU, the use of advanced support techniques, such as mechanical ventilation, increases the risk of infectious complications [87,88]. Terminally ill patients face a higher risk of healthcare-associated infections than others [89]. In particular, prolonged antibiotic use and changes in the gut microbiota promote the development of Clostridioides difficile infection [90].

These factors should be considered when making decisions about continuing aggressive treatments and providing nutritional support at the end of life, particularly in relation to the patient’s wishes and care goals.

### 5.1. Benefits of Nutritional Support

When oral nutrition provides comfort and enjoyment, it should be encouraged not only for the organoleptic pleasure it provides but also for the sense of well-being and dignity. Nutritional supplements can stimulate appetite, and even minimal or suboptimal oral nutrition may be more appropriate than CANH, which often has adverse effects. Parenteral nutrition (PN) should only be considered when other routes are not feasible or adequate [91].

Nutritional counseling (NC) aims to prevent malnutrition by providing personalized advice based on the patient’s lifestyle. Some studies have reported higher survival rates among colorectal cancer patients who received NC, as well as less severe symptoms caused by radiotherapy [92,93]. NC, together with oral nutritional supplements (ONS), may be recommended for oncology patients to achieve the necessary dietary intake [42]. Some studies have reported weight gain in individuals who received a combination of NC and ONS, as well as improved nutritional status, or at least weight maintenance [94,95].

The benefits of clinically assisted nutrition and hydration include relieving thirst, improving kidney perfusion, reducing opioid toxicity, and decreasing delirium incidents [96].

### 5.2. Limited Efficacy of Nutritional Support

Digestive tract dysfunction and anorexia contribute to resistance to standard nutritional support [32,36]. Cachexia involves the loss of muscle and fat tissue, and intravenous nutrition alone cannot prevent muscle breakdown. Therefore, treatments should target both anorexia and muscle degradation. High levels of inflammatory cytokines promote anorexia and increase energy expenditure while inhibiting muscle protein synthesis by blocking the GH/IGF-1 pathway [97]. On the other hand, inhibiting IL-1 in tumor-bearing animals improved appetite and body weight [35].

A liquid formula can be administered through tube feeding (TF) and is often used in patients who cannot swallow properly [98]. A nasogastric tube (NGT) or a percutaneous endoscopic gastrostomy (PEG) tube may also be used. Some studies have shown better preservation of the triceps fold at 6 weeks when using PEG than when employing NGT [99]. However, these effects were not sustained at 6 months, although there was a slight improvement in the QoL of PEG patients at 6 months, with no significant differences in mortality and similar complication rates between both methods [100]. PEG feeding in oncological patients may offer short-term benefits in weight maintenance and preserving QoL, but this method showed no significant differences with other alternatives (NGT or standard care) in the long run [99].

Some researchers have found no significant differences between groups of patients who did or did not receive NC [101]. Likewise, no significant differences in BMI or survival after ONS have been reported [102]. Nonetheless, the results are inconsistent and require reevaluation through larger studies to draw a definitive conclusion.

Terminally ill patients may receive nutritional support and oral hydration, but a reduction in these is part of the natural progression of the disease. As the end of life nears, feelings of hunger or thirst typically subside; thus, such patients are generally not considered candidates for CANH. Enteral feeding in their case can be complicated by aspiration syndromes, hyperhydration, edema, and diarrhea [91,103].

Gastric emptying is delayed in advanced stages of the disease because of the effects of cytokines and autonomic neuropathy. A study of 37 patients with end-stage kidney disease (ESKD) and 37 healthy controls showed that ESKD patients exhibit delayed gastric emptying [104]. Meanwhile, changes in intestinal motility (e.g., ileus and partial obstructions) occur, along with digestion and absorption disorders related to liver or pancreatic exocrine insufficiency (PEI). PEI is a complication of benign or malignant pancreatic diseases, pancreatic resections, or surgeries on the upper digestive tract, and is characterized by inadequate pancreatic enzyme activity, enzyme deficiency, and related digestive and absorption issues [105].

Opioid-induced constipation (OIC) affects 40–60% of non-cancer patients treated with opioids. OIC results from slowed gastric emptying and peristalsis, increased anal sphincter tone, and decreased secretion of bile and pancreatic fluids [106,107]. It manifests through hard stools, a feeling of incomplete evacuation, abdominal pain, and other digestive symptoms; therefore, laxatives should be administered along with opioids [108].

Care staff often face the refusal of food and hydration by cancer patients because of the latter’s loss of autonomy and psychological and physical exhaustion related to end-of-life conditions [109]. Institutionalization, through its psychological effects, discomfort, and the enforcement of a fixed meal schedule with limited food options, can trigger food refusal in individuals who may be experiencing such limited choices for the first time [110,111].

In contrast to anorexia or illness-related depression, a competent patient with a severe, advanced, debilitating disease may intentionally choose to stop eating and drinking to hasten death to avoid suffering. This is known as voluntary stopping of eating and drinking (VSED) [112,113].

### 5.3. Arguments for and Against CANH

The patient in the final phase of life, the patient with advanced dementia, the comatose patient in brain death or persistent vegetative state, and the patient who voluntarily refuses nutrition or hydration to end their life require tailored decisions regarding the initiation, maintenance, or withdrawal of CANH [114]. Such decisions are also influenced by cultural, legal, and personal perceptions, as well as the experiences and values of medical teams [115,116]. Table 1 lists the arguments for and against CANH.

## 6. Ethical Aspects

Decreased appetite and refusal to eat are natural signs of dying, indicating the end of a biological cycle. Continuing CANH support in the absence of further curative treatments can prolong the dying process without offering substantial clinical benefits, raising important ethical questions [120].

**Figure 2:**
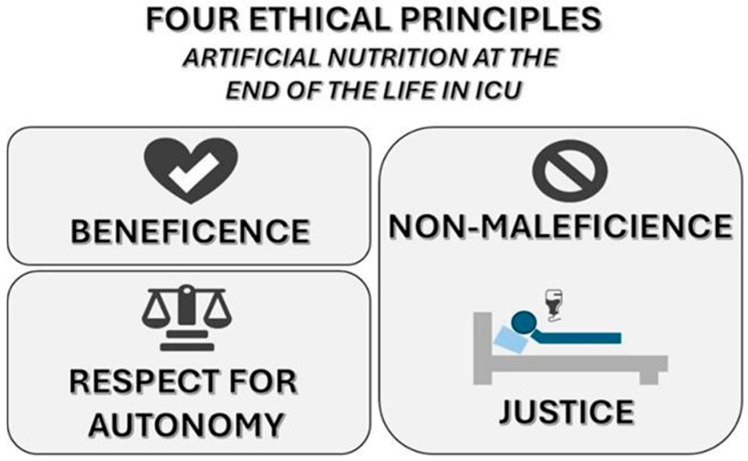
CANH at the end of life: a medical and ethical dilemma.

Beauchamp T.L. states that the four principles that serve as the foundation of medical ethics should be considered when deciding on CANH at the end of life: beneficence, non-maleficence, respect for autonomy, and justice (Figure 2) [121].

(1) The principle of beneficence calls for a careful evaluation of the potential benefits of CANH [121].
(2) The principle of non-maleficence (“no harm”) requires physicians to carefully weigh the benefits and risks of nutritional intervention [121].
(3) The principle of respect for autonomy mandates physicians to provide patients with clear information about the purpose, chances, and risks of the treatment, and a fully informed decision is then made [121].
(4) The principle of justice relates to the fair distribution of resources and treatments among patients; people with similar medical needs receive the same interventions [121].

Providing CANH when the patient voluntarily refuses it or when there is no clear benefit may be seen as a lack of respect for the patient’s will and right to self-determination [120].

In England, the Leadership Alliance for the Care of Dying People (LACDP) published the report One Chance to Get It Right (OCTGIR). The report proposes an approach for end-of-life care based on the following priorities: communicating and acting according to the patient’s wishes, involving family in decisions about medication and nutrition, and developing an individualized care plan that includes symptomatic medicines and psychological and spiritual support [121].

However, end-of-life care presents substantial ethical challenges for patients in a persistent vegetative state because these individuals are unaware of themselves or their environment. Thus, decisions regarding the continuation or withdrawal of CANH must be made within an interdisciplinary framework, involving the family and respecting any advance directives [81].

Similarly, administering CANH to brain-dead patients generates ethical concerns because the intervention does not improve the clinical condition or provide therapeutic benefits. Instead, it may artificially extend some residual bodily functions without a valid clinical purpose [130]. Therefore, CANH, whether enteral (EN) or PN, is a medical procedure that requires a solid scientific basis [14]. However, this basis is often lacking in the case of patients in the terminal phase of a disease, with advanced stages of dementia, or at the end of life [7]. According to the 2023 ESPEN Guidelines on Ethical Aspects of Artificial Nutrition and Hydration, there is no consistent evidence that enteral tube feeding in advanced dementia improves survival or quality of life. Large cohort studies show no significant differences in mortality or functional outcomes between patients receiving enteral feeding and those offered careful handfeeding (RR 1.03; 95% CI 0.92–1.15) [8].

Decisions about nutritional support in the final stages of dementia or terminal cancer raise complex ethical, logistical, and moral questions about whether to continue manual or artificial feeding because these interventions do not enhance survival or QoL [81,114]. The 2022 EAPC Recommendations on Palliative Nutrition and Hydration [131] also conclude that CANH, whether enteral or parenteral, does not significantly extend survival in patients with advanced or terminal cancer. In major studies, the median survival difference compared to standard care is less than two weeks, and symptom burden and complication rates may increase [131].

Table 2 outlines the main differences between hydration and artificial nutrition (enteral or parenteral) in relevant clinical situations. This comparison supports the clinical tools presented later in Section 10 and explains when each intervention may be appropriately matched to the patient’s needs and comfort.

### 6.1. Perception of Medical Staff Regarding EN and PN at the End of Life

Health professionals’ perception regarding CANH in end-of-life patients varies widely across institutions. According to Pala C et al., 54.3% of medical staff believe that starting CANH could improve nutritional status, with a higher percentage at the Geneva University Hospitals (66.35%) than at Inselspital (46%) and the Ticino Cantonal Hospital (47.3%). By contrast, CANH is viewed as an aggressive medical intervention by 81% of respondents at Inselspital, 80.7% at the Ticino Cantonal Hospital, and 63% at the Geneva University Hospitals. Notably, only 40% consider CANH an essential part of palliative care [115].

These results suggest that many health professionals consider CANH at the end of life an aggressive intervention. Participants highlighted the ethical dilemmas and debates surrounding this practice, particularly when deciding to withdraw or not initiate it [115]. Elise Piot et al. reported that 60% of the professionals surveyed were involved in the decision to withdraw CANH at the end of life. More than a quarter of them frequently experienced ethical dilemmas, primarily because of a lack of clear information, disagreements within the team, communication difficulties with patients and their families, and feelings of guilt and abandonment of care [116].

Many reasons may lead a physician or nutritionist to provide a patient with food in the terminal phase of a disease, even if benefits are lacking. These reasons may include avoiding a difficult discussion with the patient, protecting themselves from potential legal action, responding to family pressure, conducting research, satisfying professional curiosity, or advancing their career. Although these motivations may be legitimate, they are only justified if they primarily serve the patient’s interests and provide tangible benefits [121].

### 6.2. Informing the Family and the Patient

Open and informed discussions with the patient and/or their family about CANH at the end of life are necessary because such conversations provide them with information about the purpose of nutrition, the likelihood of success, and the associated risks. The patient’s family may thus make an informed decision whether to accept nutritional intervention [117] in sensitive clinical situations, such as a comatose state, persistent vegetative state, severe dementia, or advanced neoplasia. These exchanges help clarify values, preferences, and goals of care, avoiding decisions that do not align with the patient’s or their family’s wishes [123,124].

A key question in these situations is, When is feeding an act of love, and when does it become an extension of suffering? [132]. Many families want to “do everything they can” for their loved one. Nonetheless, force feeding in the final days of life can be unnecessary and harmful, possibly causing increased digestive discomfort or emotional distress [125]. On the other hand, comfort feeding in small amounts, with a focus on the patient’s well-being, is a way to demonstrate care and support. Forcing food when it causes distress should be avoided [15,126]. In cases where patients can no longer express their opinions, their families must be guided to make informed decisions, following the principles of palliative care [127].

Research indicates that early conversations about these issues can reduce the likelihood of choosing artificial feeding when it causes discomfort, encouraging instead compassionate and individualized care [123,124]. International guidelines recommend a personalized approach focused on the patient’s comfort and preferences, with the family participating in these decisions [10,128].

Although the term CANH is often used as a single concept, hydration and artificial nutrition are separate interventions with different physiological mechanisms, therapeutic goals, and risk profiles. Clearly distinguishing the two is vital for making appropriate decisions and communicating accurately with patients’ families.

Hydration (enteral or intravenous) aims to maintain fluid and electrolyte balance and alleviate symptoms such as thirst, opioid-induced delirium, or prerenal azotemia. However, clinical studies show that its effects are limited and variable. Some studies have reported improvements in sedation or myoclonus, while others have observed increased fluid retention (edema, ascites, pleural effusions) without clear benefits on quality of life or survival [133,134,135].

Artificial nutrition (enteral or parenteral) provides macronutrients and micronutrients, but its metabolic efficiency is reduced in advanced stages of the disease—especially in hypercatabolism, anorexia, and multiorgan failure. The ESPEN recommendations clearly emphasize that although malnutrition should be actively treated in oncology, the end of life is an exception, where the risks of artificial nutrition frequently outweigh the benefits. PN or EN should only be used selectively, in patients who are expected to die more quickly from starvation than from the progression of the disease [136,137,138].

To assist practical decision-making, short interpretative notes were added at the end of each subsection, summarizing which outcomes (survival, comfort, aspiration, and quality of life) are most affected and indicating the approximate level of certainty (high, moderate, or low). Quantitative results from key reviews and guidelines are included where available. Table 3 consolidates these findings, offering an overview of clinical settings, decisions, and their importance to outcomes.

**Table 3:** Summary of clinical contexts, decision pathways, and outcomes related to CANH at the end of life.

Population/Setting	Typical Clinical Decision	Effect on Survival	Effect on Comfort/QoL	Effect on Aspiration/Complications	Certainty of Evidence	Key References
Advanced dementia with swallowing failure	Tube feeding vs. careful hand feeding	No improvement in survival (RR 1.03; 95% CI 0.92–1.15)	No survival benefit; lower comfort	Risk of aspiration pneumonia unchanged or higher	High	[8]
Terminal cancer (cachexia, anorexia)	Parenteral or enteral nutrition vs. standard palliative care	No significant survival benefit (median difference < 2 weeks)	No consistent improvement in QoL; increased symptom burden	Higher complication rate (infection, fluid overload)	Moderate	[131]
ICU patient with poor prognosis (multi-organ failure)	Continue vs. withdraw CANH	Minimal impact on mortality in refractory cases	Possible increase in discomfort; sedation often required	High metabolic and infection risks	Moderate	[139,140]
Persistent vegetative state/minimally conscious state	Continue vs. withdraw CANH	No survival benefit; may prolong biological function without	No improvement; burdens families emotionally	Medical complications are frequent (infection, thrombosis)	Moderate–Low	[141,142]
Advanced neurodegenerative diseases (ALS, Parkinson’s)	PEG feeding vs. comfort feeding/oral support	May transiently maintain weight; no long-term survival gain	QoL may improve temporarily if aspiration is reduced	Complications related to PEG are frequent	Moderate	[143]

Note: The legal and ethical frameworks shown in this table are based on primary national legislation and official professional guidance, published or updated between 2003 and 2025, during the overall review period (2000–2025). The table is meant for guidance only; clinicians should always consult the most recent national laws and professional guidelines when making end-of-life decisions.

## 7. Legal Aspects

The Vienna Declaration: The International Declaration on the Human Right to Nutritional Care states that access to nutritional care (and thus CANH) is a fundamental human right. All persons, regardless of age, health status, or living conditions, have the right to receive adequate dietary care, which promotes their health, prevents and treats malnutrition, and enhances QoL [16].

The recommendations are aimed at all patients, including those in terminal stages, where nutrition is viewed not only as biological support but also as a form of compassionate care. The declaration also emphasizes the importance of respecting patient values, wishes, and needs, to ensure a death as free from suffering as possible [16].

In 1990, the USA enacted the Patient Self-Determination Act, which requires healthcare facilities to consider living wills and advance medical directives. The act allows individuals to choose their desired care if they become unable to make decisions because of illness. Capable individuals are guided on nutritional options (oral, parenteral, and enteral) and are encouraged to make informed choices. However, these options may change if perceptions of acceptable QoL shift [129].

Advance directives help reduce conflicts between the family and the medical team. This is critical in countries where such directives are legally recognized. For example, in the Netherlands, an act regulating the ending of life by a physician at the patient’s request in cases of unbearable suffering came into effect in 2002 [144].

Laws and regulations about assisted nutrition and hydration (ANH) vary among countries. Beyond legislation, complex cultural differences (e.g., between Western and Asian countries) create moral and ethical questions about how to treat terminally ill patients. For example, in Chinese culture, ANH is seen as a key expression of care and a moral duty of children toward their parents [145]. South Korean law considers ANH basic care, which cannot be withdrawn or replaced (National Law Information Center Act on Decisions on Life-Sustaining Treatment for Patients in Hospice and Palliative Care or at the End of Life) [145]. On the other hand, Taiwanese legislation is more permissive and allows interruption in cases of irreversible coma or vegetative state [146,147,148].

The European Society for Clinical Nutrition and Metabolism and the American Society for Parenteral and Enteral Nutrition have issued recommendations to help reduce the burden of decision-making on healthcare professionals or families. These recommendations aim to avoid unnecessary medical procedures and prevent or resolve dilemmas [15].

According to the guidelines established in 2018 by the American Academy of Neurology, CANH support in patients in a minimally conscious state or with a chronic vegetative state can only be withdrawn with the prior consent of the patient or with the approval of the legal representative [141].

The public policy documents studied mainly originate from the European Union, the United Kingdom, the United States, and Canada. These legislative and ethical frameworks can vary by administrative level (national, regional, or local), evolve, and be influenced by the specific legal and cultural context of each country.

Approaches vary considerably among countries. Table 4 summarizes the main legal and ethical frameworks governing decisions on CANH across various jurisdictions, as well as key international guidelines. Each entry includes the year, the primary legislative or professional source, the level of legal certainty, and a brief clinical interpretation.

## 8. Spiritual Food and the Human Dimension at the End of Life

Palliative care for terminally ill patients involves a multidisciplinary approach. The emotional aspect includes managing symptoms related to patients’ deep existential needs and questions [156].

Listening sincerely and empathetically to the patient is a valuable therapeutic act, as it creates a space where the patient can share spiritual issues and questions with the caregiver. Sometimes, just the presence of the clinician—not as an expert, but as a human being—is what is needed. As the Buddhist tradition says, “Don’t do something, sit there” [157].

Spiritual nourishment related to palliative care does not depend on life expectancy. In the case of terminally ill patients, this may include practices and beliefs that provide spiritual comfort, the search for an explanation for the meaning of life, and the connection with loved ones and the divine during their final journey. Addressing these aspects requires the emotional involvement of the clinician, who must remain balanced and assume the role of a “generalist of spiritual care” to manage the spiritual crisis and painful questions that accompany the end of life [157,158].

Psychologists are tasked with addressing the psychological and spiritual aspects of terminally ill patients without confusing spirituality with religion. Psychologists identify the patient’s values, needs, and suffering, along with their spiritual and religious background. Each patient is different; some may need religious care and guidance, while others may not [159].

Having a spiritual leader available can be helpful in some cases. Thus, spiritual nourishment should be included to support the mental health of the patient. Offering access to a priest or chaplain can facilitate spiritual care for both the patient and their family [160].

## 9. Limitations of the Study

We chose the narrative approach to integrate heterogeneous clinical studies, professional guidelines, and legislative texts, and selection bias is possible. Jurisdictional frameworks differ, and legal and ethical positions are evolving, which may influence the conclusions of the included studies. Therefore, the recommendations we formulate constitute only a conceptual framework, to be interpreted as general guidance adaptable to national legislation and local institutional policies.

## 10. Final Recommendations for Clinicians

Decisions about starting or stopping CANH at the end of life are among the most complex in clinical practice. Our synthesis highlights that technical feasibility does not create a moral or clinical obligation to act. A proportionate, patient-centered approach should be based on capacity assessments, a shared understanding of goals, and an acknowledgment that the mere possibility of feeding does not necessarily mean a benefit. This narrative review combines various sources, clinical evidence, professional guidance, and legal frameworks to create a structured framework for clinicians. It aims to support decisions that respect autonomy, proportionality, and the patient’s best interests.

Thus, clinicians must perform the following:

- Assess prognosis, comorbidities, and the patient’s wishes or values before starting or continuing CANH.
- Maintain an open, empathetic dialog with patients and their families to align expectations and care goals.
- Respect the patient’s autonomy by honoring their choices, even when they differ from the family’s preferences.
- Analyze risks, burdens, and benefits by considering complications (such as aspiration, infections, and discomfort) versus potential comfort or symbolic value.
- Consider all medical, psychological, social, and spiritual needs when making end-of-life decisions.
- When uncertainty occurs, a short-term trial of CANH with defined review criteria may be appropriate, provided ongoing communication with families and the multidisciplinary team persists.
- Follow institutional protocols and national laws, documenting discussions and review points.

**Figure 3:**
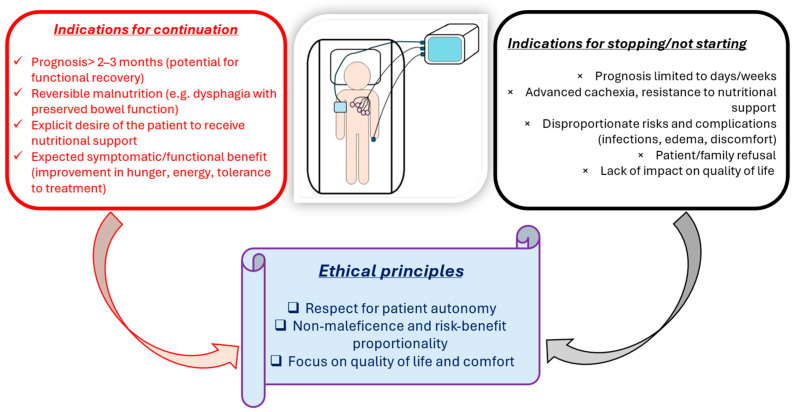
Practical criteria for nutrition at the end of life.

Figure 3 illustrates practical criteria for decisions regarding nutritional support at the end of life.

## 11. Conclusions

Decisions about starting or withdrawing CANH at the end of life are among the most complex in clinical practice and should be assessed on an individual basis, weighing potential benefits against risks, patient wishes, and family perspectives. CANH should be viewed as a therapeutic option, justified only when it serves the patient’s best interests, respects autonomy, and preserves dignity and comfort. Decisions should be guided by ethical principles and supported by clear and compassionate communication among healthcare teams, patients, and their families.

National societies and legal advisors must regulate these practices, seeking a balance between benefits and risks. This would allow medical professionals and nutrition teams to operate within legal protections.

However, many legislative and regulatory gaps remain in Romania, and the nation still requires further alignment with international legislation and standards.

## Figures and Tables

**Table 1:** Supporting arguments and counterarguments for nutrition in the final days of life.

**Nutrition of Patients at the End of Life**	
Supporting arguments [117,118,119,120,121]	Alleviates hunger and thirst, reducing patient discomfort
	Uphold the symbol of patient care, “never abandon”
	Limited support for the medical team and family during the grieving process
Counterarguments [117,118,120,121,122,123]	Rarely enhances or extends the quality of life
	Associated with higher risks (discomfort, aspiration, infections)
	Extends the dying process
	Contradicts the principles of comfort-focused palliative care
**Nutrition in comatose patients, those in a persistent vegetative state, and those in brain death**	
Supporting arguments [117,118,120,121]	Maintains essential biological functions
	Supports the family in accepting death
	Permits organ donation (in confirmed brain death)
Counterarguments [120,121,124,125]	In brain death, the patient is considered both biologically and legally dead
	In a vegetative state or deep coma, the likelihood of recovery is very low
	It causes ethical and legal confusion about the definition of death
**CANH in Patients with Advanced Dementia**	
Supporting arguments [122,123,126,127]	Prevents complications like dehydration and pressure ulcers
	It can be seen as an expression of concern for the patient
	Gives the family time to process the situation
Counterarguments [10,16,120,127,128,129]	Does not significantly improve the quality of life
	It may cause discomfort, agitation
	It is linked to a higher risk of aspiration and infection
	Artificially extends an irreversible process
**Patients who refuse CANH (do not want to live)**	
Supporting arguments [119,120]	Moral obligation to “do everything possible”
	Sustaining life might enable reconsideration of the patient’s decision
Counterarguments [16,128]	Respect for patient autonomy must be upheld
	If a capable patient refuses nutrition, their decision must be honored
	Administering CANH without the patient’s consent is unethical
	It causes unjustified psychological and physical suffering

**Table 2:** Clinical Distinctions Between Hydration and Artificial Nutrition in End-of-Life Care.

Type of Intervention	Indications	Expected Benefits	Potentially Harmful Effects	Clinical Scenarios
Hydration (oral, parenteral)	Thirst, symptoms of dehydration with prerenal azotemia, delirium from opioid toxicity, and other acute reversible conditions	Improvement in thirst and dehydration symptoms; enhancement of overall condition in carefully chosen groups	Edema, increased respiratory secretions, pulmonary congestion, and complications related to catheter placement	Dementia, advanced neuromuscular diseases, terminal cancers, and ICU patients with comfort-focused goals
Enteral nutrition (NGT/PEG)	Patients with intact gastrointestinal function for whom oral nutrition is no longer safe	May temporarily stabilize weight or slow weight loss; may help relieve hunger in some cases	Aspiration pneumonia, infections, discomfort from tube placement, and diarrhea	Neurological diseases (ALS), reversible dysphagia, and well-selected ICU cases
Parenteral nutrition	Severe gastrointestinal dysfunctions and obstructions; malabsorption syndromes	Potential symptom improvement in carefully chosen cases	Catheter infections, fluid overload, and metabolic complications	May be recommended for short periods in ICU with reversible potential; rarely considered appropriate in cases of advanced dementia or terminal cancer

**Table 4:** Legal and Ethical Frameworks Governing CANH.

Country/Guide	Primary Legal or Ethical Source (Year)	Core Recommendation/Legal Approach	Degree of Certainty	Clinical Takeaway	Additional Comments
France [149]	Claeys-Leonetti Law, Code de la Santé Publique (2016, updated May 2025)	Legalizes medical assistance in dying for incurable diseases; allows withdrawal of futile CANH under collegial medical decision.	Legislative certainty (in force)	CANH may be lawfully discontinued when deemed futile, respecting patient autonomy and prior wishes.	Allows the administration of a lethal substance at the patient’s request, or in the event of incapacity, by a medical commission
Netherlands and Belgium [150]	Termination of Life on Request and Assisted Suicide Act (The Netherlands, 2002); Belgian Euthanasia Law (2002, amended 2020)	Euthanasia and assisted suicide are allowed under strict conditions, including advanced dementia with advance directives.	Legislative certainty	Advance directives are binding; CANH withdrawal or non-initiation is ethically and legally permitted when aligned with prior wishes.	Advance directives must be respected.
United Kingdom [151]	Mental Capacity Act (2005); NICE NG31—Care of Dying Adults (2015, updated 2023)	Decisions based on “best interests”; CANH may be withdrawn if no longer beneficial; court review possible in complex cases.	Moderate certainty (jurisprudence)	Decision-making must follow capacity assessment and best-interest review; legal oversight ensures proportionality.	The multidisciplinary team decides, in complex cases, whether court involvement is necessary
Romania [152,153]	Law no. 46/2003 on Patients’ Rights; Code of Medical Deontology (Romanian College of Physicians, 2016)	Informed consent mandatory; no explicit CANH legislation; legal representative acts in cases of incapacity.	Low certainty (legislative gaps)	Ethical decisions guided by dignity and informed consent; absence of specific law may require institutional ethics input.	Decisions in the event of incapacity are made by the legal representative or relatives, with respect for the individual’s dignity.
EAPC—Palliative Care (2009) [11,131]	European Association for Palliative Care Guidelines (2009)	CANH not recommended in final days/weeks of life; sedation preferred in well-selected cases.	–	Ethical focus on comfort and proportionality rather than prolongation of life.	General Ethical Guidance
ESPEN—Nutrition in Cancer (2017) [136]	ESPEN Guidelines on Nutrition in Cancer (2017)	CANH not routinely indicated in terminal stages; considered if malnutrition is reversible and life expectancy >2–3 months.	Low	Individual assessment required; start only if benefit and comfort are expected.	Emphasis is placed on the individual assessment of each patient.
ESPEN—Practical Guidelines, Advanced Cancer (2021) [154]	ESPEN Practical Guidelines: Clinical Nutrition in Cancer (2021)	Parenteral nutrition only if prognosis >2 months and patient requests it; periodic reassessment advised.	Moderate	PN/EN indicated only for reversible situations or expected symptom relief; focus on QoL.	Care is focused on quality of life and autonomy
ASPEN—Guidelines (2016) [155]	ASPEN Clinical Guidelines for Nutrition Support in Adult Patients (2016)	PN or EN not recommended in terminally ill patients with no functional benefit.	Low	Nutrition should not be continued if it fails to improve survival, comfort, or function.	The emphasis is on the usefulness of the intervention

Note: All legal and ethical frameworks were verified using primary national legislation, official government sources, or professional guidelines, published or last updated between 2002 and 2025. The table is provided for guidance only; clinicians should always consult the most recent national laws, court rulings, and professional guidance when making end-of-life decisions regarding CANH.

## Data Availability

No new data were generated or analyzed in this study; data sharing does not apply to this article.

## Supplementary Materials

The following supporting information can be downloaded at: https://www.mdpi.com/article/10.3390/nu17233705/s1, Supplementary Figure S1. Flow diagram illustrating literature identification, screening, and inclusion process in the narrative review.

## Abbreviations

The following abbreviations are used in this manuscript:

ACS	Anorexia–cachexia syndrome
ANH	Assisted nutrition and hydration
ARC	Arcuate nucleus
ASPEN	American Society for Parenteral and Enteral Nutrition
α-MSH	Alpha-melanocyte-stimulating hormone
BBB	Blood–brain barrier
CANH	Clinically assisted nutrition and hydration
CNS	Central nervous system
COX	Cyclooxygenase
CRP	C-reactive protein
CSF	Cerebrospinal fluid
EAPC	European Association for Palliative Care
EN	Enteral nutrition
ESKD	End-stage kidney disease
ESPEN	European Society for Clinical Nutrition and Metabolism
GH/IGF-1	Growth hormone/Insulin-like growth factor-1 axis
5-HT2C	Serotonin receptor subtype 2C
IFN-γ	Interferon-gamma
IL-1	Interleukin-1
IL-6	Interleukin-6
LACDP	Leadership Alliance for the Care of Dying People
LMF	Lipid-mobilizing factor
MC4R	Melanocortin-4 receptor
NC	Nutritional counseling
NGT	Nasogastric tube
NPY	Neuropeptide Y
OCTGIR	One Chance to Get It Right
OIC	Opioid-induced constipation
ONS	Oral nutritional supplements
PEG	Percutaneous endoscopic gastrostomy
PEI	Pancreatic exocrine insufficiency
PGE2	Prostaglandin E2
PN	Parenteral nutrition
POMC	Proopiomelanocortin
QoL	Quality of life
SNPs	Single-nucleotide polymorphisms
TF	Tube feeding
TNF-α	Tumor Necrosis Factor-alpha
TP53, MYC, HIF1A	Tumor driver genes (oncogenes and transcription factors)
VMH	Ventromedial hypothalamic nucleus
VSED	Voluntary stopping of eating and drinking
