# Supplementary figures and images for: Nutrition and Hydration at the End of Life in Intensive Care and General End-of-Life Care Settings: Balancing Clinical Evidence, Patient-Centered Care, and Ethical and Legal Principles—A Narrative Review
Source: Nutrients. 2025 Nov 26;17(23):3705. doi: 10.3390/nu17233705 (PMC12693952; doi:10.3390/nu17233705)

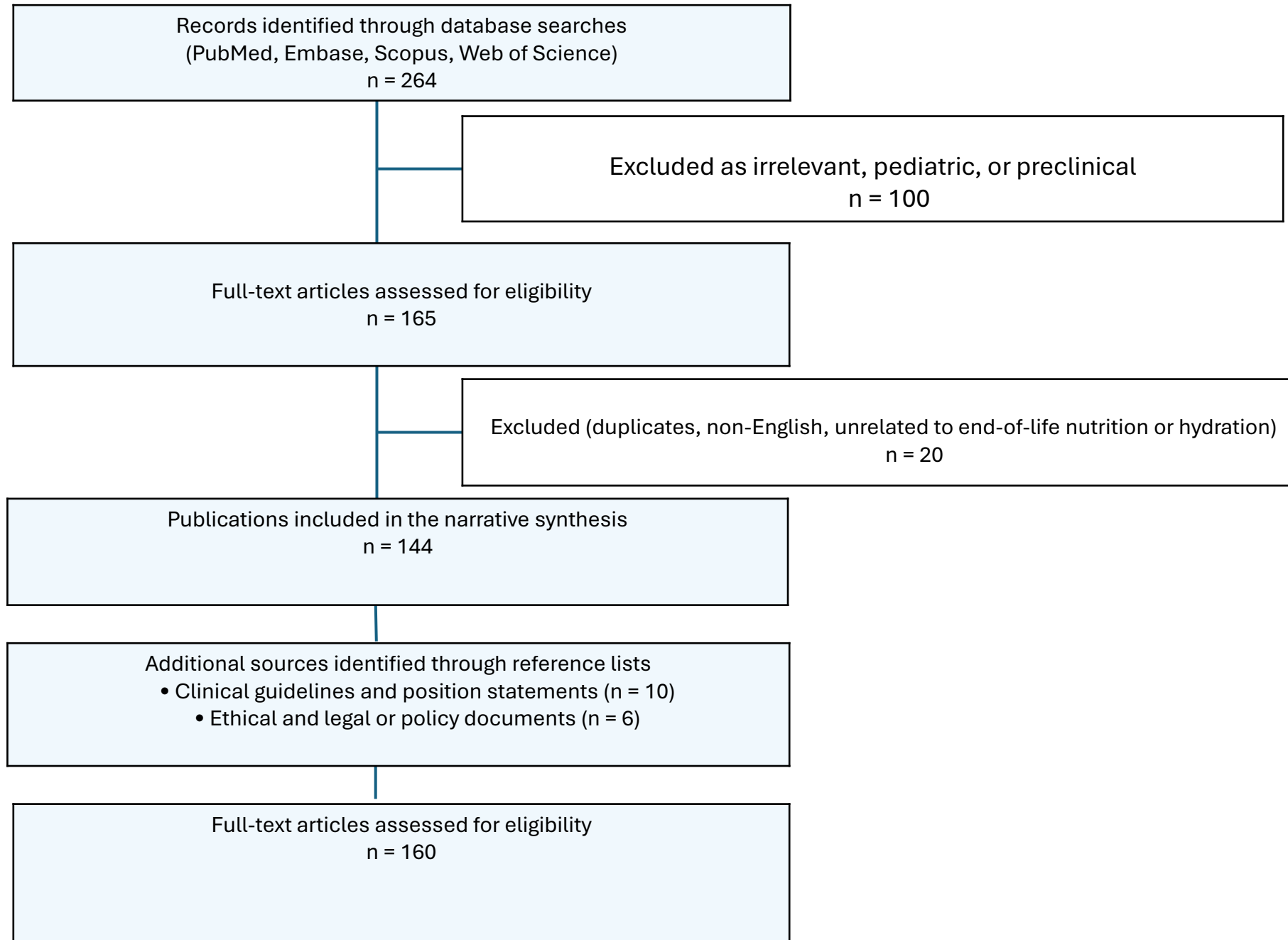

Supplement: Supplementary file 1 [file nutrients-17-03705-s001.zip › nutrients-3939785-supplementary.pdf]
